# Evidence integration and bridging methodologies for high-risk and innovative medical devices and *in vitro* diagnostic medical devices: a scoping review

**DOI:** 10.3389/fmed.2026.1917208

**Published:** 2026-08-28

**Authors:** Nensi Bralić, Ružica Bandić, Miro Vuković, Danira Matijaca, Renata Valsami, Panagiotis Zoumpoulakis, Kalle Schnitzer, Mari Levula, Risto Roine, Antti Viitala, Antti Pihlava, Olivia McDermott, Ana Marušić

**Affiliations:** 1 Department of Research in Biomedicine and Health, University of Split School of Medicine, Split, Croatia; 2 Central Medical Library, University of Split School of Medicine, Split, Croatia; 3 Greek Patients’ Association, Athens, Greece; 4 Clinipower Finland Oy, Savonlinna, Finland; 5 SGS Fimko Ltd., Helsinki, Finland; 6 College of Science & Engineering, University of Galway, Galway, Ireland

**Keywords:** adaptive trial design, Bayesian method, bridging studies, evidence integration, *in vitro* diagnostics (IVD), medical device (MD), real-world data, regulatory science

## Abstract

**Background:**

High-risk and innovative medical devices, as well as *in vitro* diagnostic medical devices, often pose challenges for conventional clinical evaluation because randomized clinical trials may be difficult to conduct. As a result, methodologies for integrating heterogeneous evidence and conducting bridging studies are becoming increasingly important in regulatory decision-making and health technology evaluation.

**Objective:**

To map methodologies for evidence integration and bridging studies in high-risk and innovative medical devices and *in vitro* diagnostic medical devices, and to identify key methodological and regulatory challenges.

**Methods:**

A scoping review following Joanna Briggs Institute guidance was conducted using peer-reviewed and grey literature published from 2017 onward. Bibliographic databases, industry resources, and preprint platforms were searched. Included records addressed methods for comparing or integrating evidence for medical devices and *in vitro* diagnostic medical devices. Data extraction and synthesis were supported by artificial-intelligence-assisted analysis, with human verification.

**Results:**

Nineteen studies were included. Identified methods included Bayesian evidence synthesis, adaptive trial designs, indirect comparisons, propensity score methods, real-world data integration, linked evidence frameworks, in silico approaches, and bridging studies. These methods support evaluation when conventional randomized clinical trials are limited or infeasible. Major challenges included evidence heterogeneity, bias, limited methodological standardization, and variable regulatory acceptance, particularly for *in vitro* diagnostic medical devices and digital technologies.

**Conclusion:**

Evidence integration methodologies are increasingly important in the evaluation of innovative medical devices and *in vitro* diagnostic medical devices. However, clearer methodological standards and harmonized guidance are still needed to support consistent and reliable use of heterogeneous evidence.

**Systematic review registration:**

The scoping review was registered with the Open Science Framework (OSF). The Unique Identifier is 10.17605/OSF.IO/XNBSP, and the registration is publicly available at https://doi.org/10.17605/OSF.IO/XNBSP.

## 1 Introduction

Generating robust clinical evidence for high-risk and innovative medical devices (MDs) and *in vitro* diagnostic medical devices (IVDs) remains a major methodological challenge in contemporary healthcare research and regulation (1, 2). Unlike pharmaceuticals, MDs and IVDs often undergo iterative modifications throughout their lifecycle, while their performance may depend on clinical context, operator expertise, and learning curves (1, 2). High-risk and innovative MDs and IVDs are particularly important because of their potential impact on patient outcomes and healthcare delivery (2, 3). Due to their complexity and rapid technological evolution, these technologies require robust evidence to demonstrate safety, effectiveness, and performance throughout their entire life cycle (1, 4).

The implementation of the Medical Device Regulation (EU MDR 2017/745) and the *In Vitro* Diagnostic Medical Device Regulation (EU IVDR 2017/746) has substantially strengthened evidence requirements for MDs and IVDs in the European Union (EU) (3, 5, 6). These regulatory frameworks introduced reinforced requirements for clinical and performance evaluation, post-market surveillance (PMS), transparency, and traceability, while emphasizing the generation of continuous evidence throughout the product lifecycle (3–6). Conventional evidence-generation approaches may not adequately address the specific characteristics of high-risk and innovative devices (1, 2).

Traditionally, evidence-based medicine (EBM) has relied primarily on randomized controlled trials (RCTs) as the highest level of evidence (7). However, decision-making in healthcare increasingly requires broader approaches that integrate multiple sources of information, including observational studies and real-world evidence (RWE) (8, 9). Growing recognition of the limitations associated with relying solely on traditional trial designs has led to increasing interest in more comprehensive evidence-generation strategies (2, 10).

Alternative and complementary evidence sources, such as real-world data (RWD), patient registries, and post-market surveillance data, have gained increasing importance (11). Well-designed registries and coordinated registry networks can provide valuable evidence when randomized trials are impractical or insufficient due to ethical, technical, or logistical constraints (11). However, integrating evidence from diverse sources into regulatory and health technology assessment (HTA) decision-making requires methodologically robust approaches that account for differences in data quality, bias, relevance, and applicability (9, 12).

Evidence integration can be understood as the process of combining multiple sources of evidence to support more comprehensive and meaningful decision-making (8, 12). Methodological approaches include narrative synthesis, thematic synthesis, and increasingly advanced quantitative approaches for integrating heterogeneous forms of evidence (12, 13).

Bridging studies represent an important methodological strategy within this broader framework of evidence integration (14). They facilitate the use of existing evidence in new contexts by enabling extrapolation from previous studies, related products, or alternative populations while reducing unnecessary duplication of research efforts (14).

Advanced statistical methods have further expanded opportunities for evidence integration. Bayesian approaches enable formal incorporation of prior information with newly generated data to improve estimation and decision-making (15). Bayesian dynamic borrowing (BDB) adaptively incorporates external evidence to improve efficiency and potentially reduce sample-size requirements, although its validity depends strongly on comparability between data sources (16).

Additional challenges remain in implementing integrated evidence-generation strategies. Coverage with evidence development (CED) schemes can support early patient access while simultaneously generating additional evidence, but their implementation remains complex and heterogeneous across healthcare systems (9). Challenges include study design considerations, stakeholder coordination, management of device modifications over time, and substantial variability in evidence-generation practices across jurisdictions (9, 17).

Emerging technologies, including digital and artificial intelligence-based medical devices, introduce further methodological concerns related to transparency, continuous validation, and data quality (10).

Despite increasing reliance on multiple evidence sources and evolving methodological approaches, evidence generation for high-risk and innovative MDs and IVDs remains fragmented and insufficiently standardized (2, 10, 17). In particular, a comprehensive understanding of existing methodologies for evidence integration and bridging studies in this field remains limited. Therefore, the aim of this scoping review is to map and analyse existing methodologies for evidence integration and bridging studies related to high-risk and innovative MDs and IVDs, and to identify key methodological challenges and gaps in current practice. To provide a broader context, relevant international methodological and regulatory perspectives were also considered alongside the EU MDR/IVDR framework.

## 2 Methods

This scoping review was conducted in accordance with the methodological guidance provided by the Joanna Briggs Institute (JBI) Manual for Evidence Synthesis (12). The protocol was registered at the Open Science Framework (OSF) (18). This scoping review was conducted within the framework of the EU4MEDTECH project and followed the same protocol and literature search strategy as a previously published scoping review on methodologies for generating and evaluating clinical and performance evidence for high-risk and innovative medical devices and *in vitro* diagnostic medical devices (19). A common protocol and search strategy were developed to capture the full spectrum of methodological approaches relevant to the regulatory assessment of these technologies, including evidence generation, evaluation, integration, and bridging. As these domains represent interconnected components of the regulatory evidence framework, a single comprehensive search was considered the most appropriate approach to ensure consistency, transparency, and completeness of evidence identification. While the previous review focused on methodologies for evidence generation and evaluation, the present review addressed the distinct objective of examining approaches to evidence integration and bridging studies. Accordingly, study selection, data extraction, and synthesis were undertaken in line with this specific review objective.

### 2.1 Data sources

Records from both peer-reviewed and grey literature addressing methodologies for integrating clinical and performance evidence were included, with particular emphasis on high-risk or innovative MDs and IVDs. Relevant sources were identified through comprehensive searches conducted across multiple bibliographic databases, including PubMed, Scopus/Embase, and Web of Science. In addition to academic databases, resources from professional and industry organizations, including MedTech Europe, JAMS, and FDB Prizm, as well as the EU–funded research portal CORDIS, were systematically searched. To ensure the inclusion of recent and emerging evidence, preprint platforms not yet fully indexed in conventional databases were also reviewed, including PrePubMed, OSF Preprints, and search.bioPreprint. Journals with a specific focus on HTA were also systematically examined to identify relevant methodological literature. Eligible sources comprised original research articles, systematic reviews, opinion papers, and regulatory or methodological guidance documents.

### 2.2 Search strategy

A comprehensive search of both peer-reviewed and relevant grey literature was conducted on 30 January 2025 using a search strategy developed in collaboration with an experienced librarian (DM). Due to the complexity and variation in terminology in this field, the approach prioritized sensitivity over specificity to include a broad range of evidence. Peer-reviewed publications were identified through systematic searches of bibliographic databases, while grey literature was sourced from targeted resources.

Because a large amount of new material has been published recently, the search was updated on 11 November 2025 to include the most recent evidence. The full search strategy is provided in Supplementary file 1.

### 2.3 Inclusion and exclusion criteria

Original research articles, systematic reviews, opinion papers, theses, and relevant methodological or regulatory guidance documents were included. No language restrictions were applied during the selection process. Records were restricted to those published on or after 5 April 2017, corresponding to the date of adoption of the EU MDR and EU IVDR. This time restriction was applied because the aim of the review was to synthesize evidence relevant to the current EU regulatory framework governing medical devices, making studies published before the adoption of these regulations less directly applicable to the review objectives.

Records were eligible for inclusion if they explicitly described, evaluated, applied, or provided methodological guidance on approaches for evidence synthesis, indirect treatment comparisons, or the integration of multiple evidence sources in the assessment of MDs or IVDs. Eligible methodological approaches included standard meta-analysis methods, such as frequentist methods (e.g., inverse-variance, Mantel–Haenszel, Peto, and beta-binomial models), Bayesian methods, and methods for meta-analyses involving a small number of studies (e.g., the Knapp–Hartung method). Records were also eligible if they addressed indirect comparison methodologies, including the Bucher method, network meta-analysis, Matching-Adjusted Indirect Comparison (MAIC), Simulated Treatment Comparison (STC), or proportional hazards–based comparative approaches. In addition, records were included if they described methodological approaches for integrating different evidence sources, including clinical trial data, performance studies, and real-world data (RWD), in the evaluation of MDs or IVDs. Records were excluded if they were not relevant to MDs or IVDs. Studies focused exclusively on pharmaceuticals, general clinical care, or other health technologies outside the scope of MDs/IVDs were excluded, as were those containing only incidental references to these technologies. Documents limited to descriptive summaries of regulatory frameworks, without providing methodological insight, analysis, or critical discussion, were also excluded. Any records published prior to 5 April 2017 were excluded, regardless of source.

### 2.4 Study selection

All references identified through the search strategy were imported into Rayyan, an online tool for managing systematic reviews (20). The screening process was conducted in two sequential phases. In the first phase, titles and abstracts were screened to identify potentially relevant records. In the second phase, the full texts of all potentially eligible records were retrieved and assessed in detail against the predefined inclusion and exclusion criteria. A relatively large number of records underwent full-text assessment because information available at the title and abstract stage was often insufficient for confident eligibility decisions. Many records provided limited methodological detail or represented diverse publication types, including reviews, commentaries, and guidance documents. To reduce the risk of excluding potentially relevant sources, a deliberately inclusive screening approach was applied during study selection. This approach aligned with the broader scoping review objective of comprehensively mapping heterogeneous evidence (12).

Screening was performed independently by 12 reviewers (AM, NB, RB, MV, RV, PZ, ML, RR, AV, AP, KS, OM), working in pairs. Inclusion required agreement between both reviewers. Any disagreements were discussed and resolved by the lead research team (AM, NB, RB, MV).

### 2.5 Data extraction

A structured data extraction form was developed in a spreadsheet. Each included record was assigned a unique Study ID. For all eligible studies, bibliographic and methodological information was extracted, including study title, first author, year of publication, year range of included studies (for reviews), and study design.

Data extraction was conducted in two sequential phases. In the first phase, studies were categorized according to the topics they addressed in sufficient detail. This process was performed by four reviewers (AM, NB, RB, MV), working in pairs. Categorization was guided by predefined thematic categories derived from the inclusion criteria: methods for comparing evidence and integrating different types of evidence. Each study could be assigned to one or both categories, depending on its scope and content. Independent assessments were conducted to determine whether a study addressed a given category in sufficient depth to justify further extraction and analysis.

In the second phase, an Artificial Intelligence (AI)-assisted approach to in-depth topic extraction was applied. Given the volume of studies and the complexity of the data, an AI tool (21) was used to support the extraction process, which improved efficiency and consistency compared to manual extraction alone. AI-assisted approaches have increasingly been incorporated into systematic review workflows, including study screening, full-text analysis, and evidence synthesis, particularly in reviews involving large and heterogeneous bodies of literature (22–24). The process was conducted in three iterative steps. First, prompt templates were developed and refined through testing on a sample of studies to ensure clarity and relevance. Second, for each included study and its assigned category, GPT-4o (21) was used to extract structured information across four predefined dimensions: (i) overview of methods, (ii) purpose and application in the regulatory context, (iii) limitations and challenges, and (iv) variability and gaps in the literature. The use of predefined prompts and extraction domains was intended to enhance the consistency and comparability of outputs across studies. Third, the extracted outputs were synthesized across studies and categories to produce structured summaries. Prompts used for the AI-assisted extraction and synthesis process are available in Supplementary file 2.

All AI-generated outputs were subsequently reviewed, verified, and, where necessary, corrected by the authors to ensure accuracy, precision, and appropriate interpretation. Human oversight was applied throughout the extraction and synthesis process, and all AI-generated outputs were independently reviewed by the authors against the original articles to minimize the risk of inaccuracies, misinterpretations, or omissions associated with AI-generated content. Final summaries for each category were developed by grouping findings across studies and are presented in the Results section. The resulting groupings represent thematic syntheses of methodological approaches identified in the literature and are intended as conceptual categories rather than hierarchical classifications or recommendations for specific regulatory scenarios.

## 3 Results

### 3.1 Study selection process

The initial database search identified 15,679 records. After removing 1,420 duplicate records, 14,259 records remained and were screened at the title and abstract levels. Following screening, 859 full-text articles were assessed for eligibility, and 17 studies were included for data extraction.

Additionally, records were identified through other methods, including the project database CORDIS and industry and professional databases. A total of 1,323 records were screened, with 75 full-text articles assessed for eligibility. Of these, two studies met the inclusion criteria and were included in the review.

**Figure 1:**
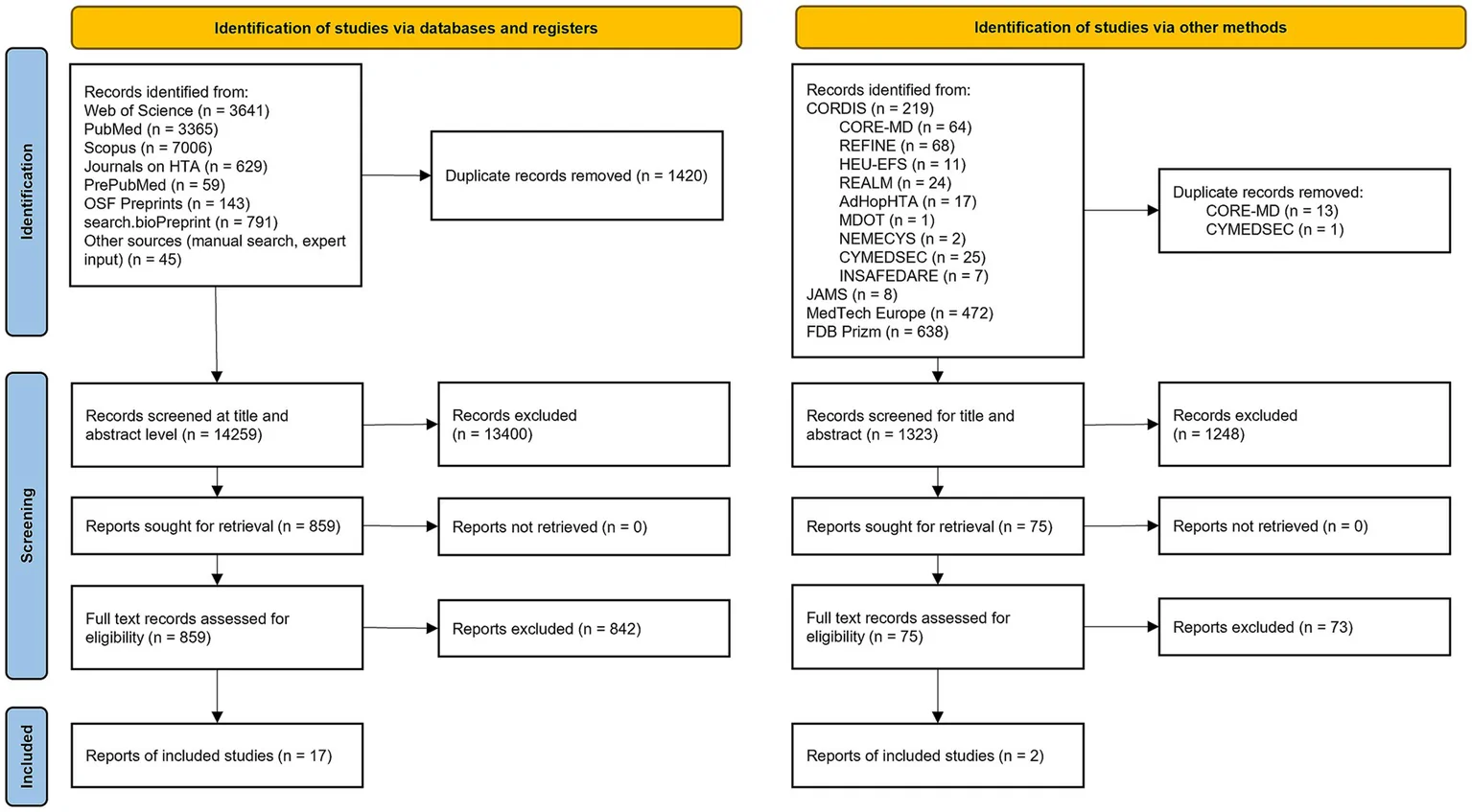
PRISMA flowchart.

In total, 19 studies were included in the final analysis (15, 25–42) (Figure 1 and Table 1). They were published between 2017 and 2024, during a period when methods in this field were changing after the introduction of the EU MDR and EU IVDR.

**Table 1:** Characteristics of studies included in the scoping review.

No.	Author(s) and Year	Title	DOI	Publication type	Region/regulatory context	Main methodological topic
1	Cao et al. (15)	Bayesian approach for design and analysis of medical device trials in the era of modern clinical studies	10.1515/mr-2023-0026	Review article	USA (FDA); international	Bayesian methods for medical device trials, adaptive designs, information borrowing
2	Chiaruttini et al. (26)	Bayesian dynamic borrowing in group-sequential design for medical device studies	10.1186/s12874-025-02520-6	Original research article	International	Bayesian dynamic borrowing, group-sequential design, historical controls
3	de Pouvourville et al. (35)	Real-world data and evidence in health technology assessment: when are they complementary, substitutes, or the only sources of data compared to clinical trials?	10.1016/j.therap.2022.11.001	Original research article	France/European HTA context	Real-world data and evidence in health technology assessment
4	Douze et al. (41)	Evaluations of medical device usability during clinical investigations: a scoping review of clinical study protocols	10.1080/17434440.2024.2378093	Scoping review	International	Integration of usability assessments into clinical investigations
5	Ferrante di Ruffano et al. (42)	Health technology assessment of diagnostic tests: a state of the art review of methods guidance from international organizations	10.1017/S0266462323000065	Review article	International HTA context	HTA methodology for diagnostic tests; evidence linkage
6	Jang (30)	Computer-Aided Clinical Trials for Medical Devices	N/A	PhD dissertation	USA	Computer-aided clinical trials, virtual cohorts, modelling and simulation
7	Jørgensen (37)	Regulatory requirements for companion diagnostics and drug-diagnostic codevelopment in the United States	10.1016/B978-0-12-813539-6.00016-X	Book chapter	USA (FDA)	Companion diagnostics and drug–diagnostic co-development
8	Li and Easton (34)	Practical considerations in clinical strategy to support the development of injectable drug-device combination products for biologics	10.1080/19420862.2017.1392424	Review article	International/regulatory	Bridging strategies for injectable drug–device combination products
9	Lu et al. (33)	Good statistical practice in utilizing real-world data in a comparative study for premarket evaluation of medical devices	10.1080/10543406.2019.1632880	Review/Guidance article	USA (FDA)	Good statistical practice for RWD in premarket medical device evaluation
10	MedTech Europe Clinical Evidence Working Group (28)	Clinical Evidence Requirements under the EU In Vitro Diagnostics Regulation (IVDR)	N/A	Guidance document	European Union (IVDR)	Clinical evidence requirements for *in vitro* diagnostic devices
11	Mühlbacher and Juhnke (27)	Benefit Assessment for Examination and Treatment Methods with Medical Devices of High Hisk: Trade-off between Patient Benefit, Evidence and Access	10.1055/s-0043-112742	Review article	Germany/European Union	Benefit assessment, adaptive study designs, MCDA
12	Pazart et al. (40)	Threats and Opportunities for the Clinical Investigation of high-risk Medical Devices in the context of the new European Regulations	10.5220/0010382902740284	Conference paper	European Union (MDR)	Clinical investigation of high-risk medical devices
13	Schnell-Inderst et al. (32)	Report on Study design Recommendations in Guidance Documents for high-risk Medical Devices	N/A	Project deliverable	International	Guidance on study design for high-risk medical devices
14	Shimazawa and Ikeda (38)	Regulatory requirements for companion diagnostics—Japan	10.1016/B978-0-12-813539-6.00019-5	Book chapter	Japan (PMDA)	Companion diagnostics regulation
15	Song et al. (39)	Statistical considerations for some issues in clinical bridging studies evaluating companion diagnostic devices	10.1080/10543406.2023.2220398	Original research article	USA (FDA)	Statistical methodology for clinical bridging studies of companion diagnostics
16	Stüdeli and Hochberg (29)	Clinical Usability Studies—Clash of Cultures? Study Design Proposal from Lessons Learned	N/A	Conference paper	International	Integration of usability and clinical investigations
17	Vidal et al. (31)	Contribution of Methodologies Adapted to Clinical Trials Focusing on High Risk Medical Devices	10.5220/0009374503370343	Conference paper	European Union	Adaptive and Bayesian methodologies for high-risk medical device trials
18	Ziegler et al. (25)	A Modular Approach to Combine Postmarket Clinical Follow-Up Studies and Postmarket Surveillance Studies	10.1055/s-0041-1735165	Original research article/short paper	European Union (MDR)	Modular integration of PMCF and post-market surveillance
19	Zisis et al. (36)	Real-world data: a comprehensive literature review on the barriers, challenges, and opportunities associated with their inclusion in the health technology assessment process	10.3389/jpps.2024.12302	Systematic review	International	Real-world data and evidence in HTA

A broad range of evidence sources was included to capture methodological, conceptual, and regulatory perspectives relevant to evidence integration and bridging methodologies for high-risk and innovative MDs and IVDs. Consistent with the scoping review methodology, all sources were analysed using a structured data-charting and narrative synthesis approach, without applying formal evidence hierarchies or weighting by study type (12). Different source types contributed complementary perspectives to the synthesis: empirical studies and evidence syntheses primarily informed methodological aspects, whereas project-based and non-empirical sources provided conceptual, implementation, and regulatory insights. This diversity may itself reflect the evolving and methodologically heterogeneous nature of the field.

**Figure 2:**
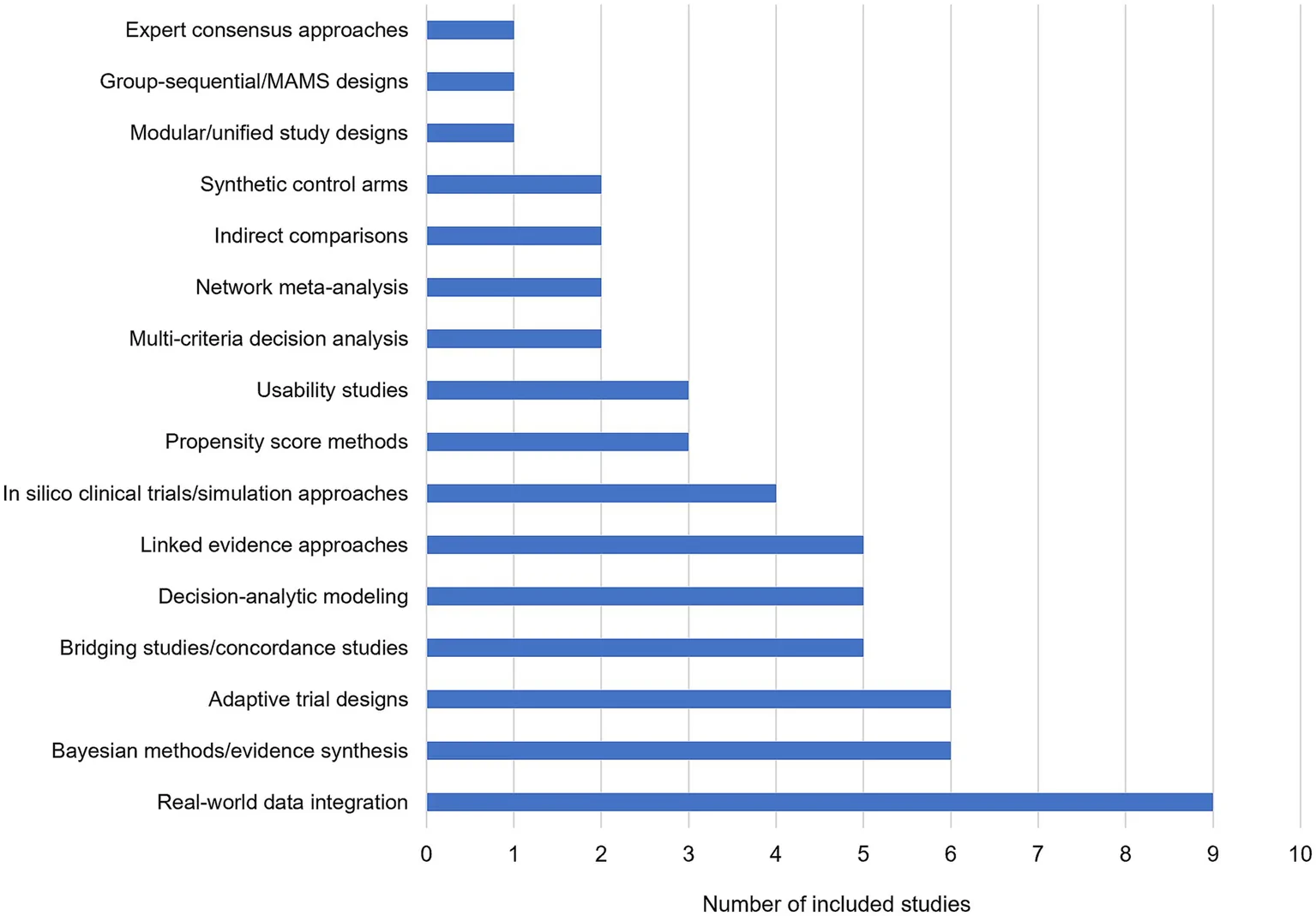
Frequency of methodological approaches identified across included studies. The figure presents the number of included studies that report methodological approaches to comparing and integrating evidence for high-risk medical devices and *in vitro* diagnostic medical devices. Individual studies could contribute to more than one methodological category.

The distribution and frequency of methodological approaches related to evidence comparison and evidence integration across the included studies are presented in Figure 2. Individual studies could contribute to more than one methodological category.

### 3.2 Approach to data analysis

To organize the findings, the included studies were analysed using two main categories based on the inclusion criteria: (i) methods for comparing evidence and (ii) the integration of different types of evidence. Some studies covered both categories and were analysed in both contexts. The results in the following sections are presented by category, focusing on methodological features, practical use, and regulatory relevance.

### 3.3 Methods for comparing evidence

#### 3.3.1 Overview of methods

The included studies describe a wide range of methodological approaches used to compare clinical and performance evidence for the regulatory evaluation of high-risk and innovative MDs and IVDs.

Bayesian methods include approaches such as hierarchical models, meta-analytic-predictive (MAP) and robust MAP (RMAP) priors, multisource exchangeability models (MEMs), self-adaptive mixture (SAM) priors, propensity score-integrated priors, and different types of power priors. These methods are used to combine evidence from multiple sources, borrow information across studies, and support comparisons when direct evidence is limited or heterogeneous. Bayesian approaches are particularly common in the evaluation of high-risk and innovative MDs, including cardiovascular devices and drug-eluting stents, because they can be adapted to multiregional and subgroup analyses (15, 26, 31, 40).

Adaptive and group-sequential trial designs, including multi-arm multi-stage (MAMS) designs, enable interim analyses, early stopping of trials, and adjustments to sample size during the study. These approaches are considered efficient and flexible, especially for rapidly evolving or operator-dependent devices, where conventional RCTs may be difficult to conduct (26, 31, 40).

Meta-analyses and systematic reviews, including Bayesian meta-analyses, are commonly used to combine findings from multiple studies, summarise safety and performance data, and address differences between studies. These approaches are particularly useful when direct comparative evidence is limited. However, strict methodological criteria are required when they are used to support claims of equivalence (31, 32).

Indirect comparisons and network meta-analyses are recognized as alternative approaches for comparative evaluation when direct head-to-head trials are not feasible. These methods are particularly useful for comparing new devices with several existing alternatives. However, clear recommendations and detailed guidance on their use remain limited (32).

Bridging studies are used to apply clinical findings from one setting, population, or version of a device to another. They support regulatory evaluation without requiring repeated full clinical investigations (32).

Propensity score methods and RWD approaches are commonly used in non-randomized comparative studies, particularly when RCTs are not feasible. These methods aim to reduce baseline differences between groups and approximate randomization, thereby supporting the generation of premarket evidence for MDs (33, 40).

In silico and simulation-based methods, including in silico clinical trials (ISCTs) and computer-aided clinical trial (CACT) frameworks, combine real and simulated patient data to assess device safety and performance. These approaches are especially relevant for complex devices, such as implantable cardioverter defibrillators (ICDs) (30, 40).

Linked evidence approaches and decision-analytic modelling are mainly applied to diagnostic devices. They combine evidence from different sources, such as test accuracy, clinical management, and treatment outcomes, when direct end-to-end evidence is unavailable. These approaches are often supported by structured frameworks developed by HTA bodies (42).

Comprehensive cohort studies, expertise-based trials, and cluster trials are described as alternative study designs that can improve feasibility and acceptability while indirectly supporting comparative evaluation through greater generalisability and reliability (31).

**Figure 3:**
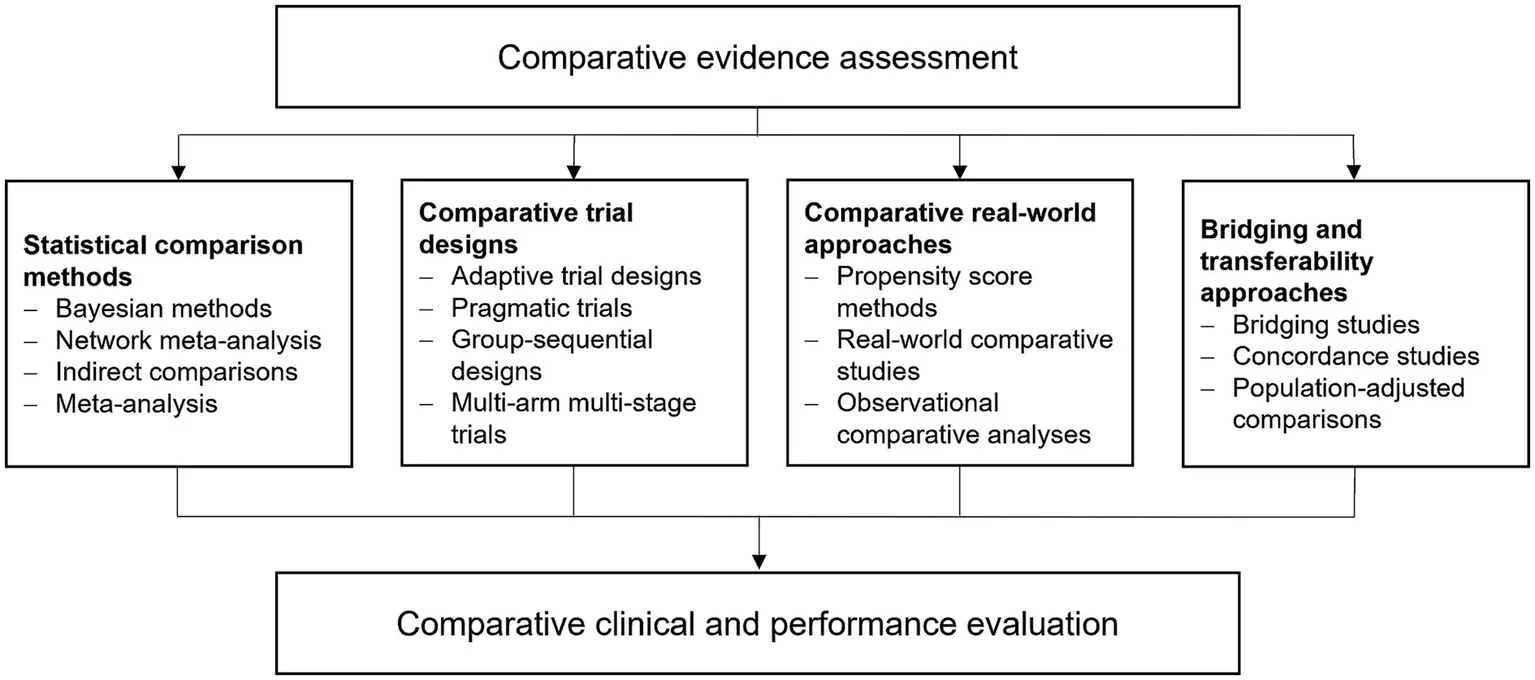
Comparative evaluation of evidence in high-risk medical devices and *in vitro* diagnostic medical devices.

The identified methodological approaches for comparing evidence were grouped into four main categories: statistical comparison methods, comparative trial designs, comparative real-world approaches, and bridging and transferability approaches. A conceptual overview of these categories and their role in comparative evaluation is presented in Figure 3.

#### 3.3.2 Purpose and regulatory application

Regulatory guidance issued within the EU MDR framework and by the Food and Drug Administration (FDA) increasingly includes references to comparative methodological approaches, particularly Bayesian methods and adaptive trial designs. The reviewed literature indicates growing regulatory acceptance of these approaches and suggests that, in some circumstances, they may be particularly relevant for high-risk and innovative medical devices (15, 26, 31, 40). For example, the FDA has issued guidance on the use of Bayesian statistics for medical device clinical trials, and Bayesian methods have subsequently been incorporated into numerous FDA-approved medical devices (15).

For IVD evaluation, regulators and HTA organizations have recognized the use of linked evidence approaches and decision-analytic modelling. However, concerns remain regarding the integration of heterogeneous evidence, highlighting the need for greater methodological consistency and standardization (42).

The use of RWD also appears to be gaining broader acceptance, particularly when supported by rigorous comparative methods that address bias and confounding, such as propensity score–based approaches (15, 30, 32, 33, 40).

#### 3.3.3 Limitations and challenges

A major concern is heterogeneity and inconsistency. Combining evidence from different study designs, populations, or device versions may introduce bias and reduce comparability. This issue is especially relevant for diagnostic technologies, where guidance on resolving inconsistencies between direct and indirect evidence remains limited (15, 31, 32, 42).

Challenges related to regulatory acceptance are also reported. Although Bayesian and in silico methods are increasingly recognized, detailed methodological guidance and regulatory acceptance are still insufficient for some approaches, including network meta-analyses and linked evidence models (31, 40, 42).

Bias and confounding remain important concerns in non-randomized studies using RWD. These study designs require careful adjustment for confounding factors and thorough assessment of bias (33, 40).

The complexity and validation requirements of advanced methods such as MEMs, SAM priors, and CACT frameworks may restrict their wider use. These approaches require advanced statistical expertise and validation, which can limit their implementation in routine practice (15, 26, 30, 31).

#### 3.3.4 Variability and gaps in the literature

The literature shows considerable variability in the level of detail and clarity in describing comparative methodologies. Some studies present detailed statistical protocols, especially for Bayesian and propensity score methods, while others mention these approaches only broadly and without specific methodological guidance (15, 26, 30, 31, 33).

Variability is also seen in device-specific guidance. These methods are more commonly applied to high-risk and innovative MDs, whereas their application in the evaluation of IVDs is discussed less frequently. An important exception is linked evidence approaches for diagnostic tests (42).

Regulatory perspectives also vary by methodology and device type. Although Bayesian and adaptive designs are increasingly recognised, detailed guidance on the integration of indirect or heterogeneous evidence, particularly for IVDs, is still limited (15, 26, 30–33, 40, 42).

In addition, several methodological areas remain underexplored, including network meta-analyses, bridging studies, and approaches to resolving inconsistencies between direct and indirect evidence. The integration of heterogeneous data sources and the implementation of complex comparative frameworks, especially for diagnostic technologies, continue to represent developing challenges (31, 32, 40, 42).

### 3.4 Integration of different types of evidence

#### 3.4.1 Overview of integration methods

The reviewed literature describes a wide range of methodological approaches for integrating clinical and performance evidence in evaluating high-risk or innovative MDs and IVDs. Integration is the systematic combination of data from multiple sources, including clinical trials, RWD, registries, modelling approaches, literature, expert input, and usability studies, to create a coherent, comprehensive evidence base for regulatory submissions.

Modular and unified study designs allow Post-Market Clinical Follow-up (PMCF) and PMS activities to be combined within a single protocol. These designs support the integration of RWD, functional assessments, and patient-reported outcomes, while remaining aligned with regulatory requirements, such as the EU MDR and ISO 14155 (25).

Bayesian evidence synthesis combines prior knowledge from historical data, expert opinion, or literature with new clinical study data through hierarchical models, adaptive designs, and propensity score-integrated priors to formally combine heterogeneous evidence (15, 30, 40).

Adaptive and pragmatic trial designs are developed to reflect the iterative nature of device development. These include Bayesian adaptive, sequential, and MAMS trials, which allow interim analyses and the inclusion of data from different sources (15, 27, 40).

Linked evidence approaches and decision-analytic modelling are particularly relevant for diagnostics. These methods use decision-analytic models to combine indirect evidence across the test-treatment pathway by integrating test accuracy, changes in clinical management, and outcomes (42).

Clinical usability studies combine clinical and usability endpoints. These hybrid studies integrate information from usability testing, interviews, device use logs, clinical trials, and questionnaires to provide a broader understanding of device performance and user interaction (29, 40, 41).

CACTs combine in silico modelling, virtual cohorts, and synthetic control arms with empirical data to support benefit–risk assessment when RCTs are not feasible (30, 40).

Risk-based hierarchical approaches, mainly applied in drug-device combinations, combine analytical, nonclinical, and clinical evidence, with the level of methodological rigor depending on the risk and timing of product development changes (34, 37).

Bridging and concordance studies are used in companion diagnostics to transfer evidence from prototype assays used in trials. These studies generally rely on stored samples and statistical modeling to combine analytical and clinical validation data (37–39).

Multi-criteria decision analysis (MCDA) integrates different clinical and patient-centred outcomes. It supports benefit assessment by weighting patient preferences together with clinical endpoints (27, 35).

Systematic literature reviews and expert consensus are commonly used to support scientific validity and performance evaluations, particularly when direct clinical evidence is unavailable or limited (28, 40).

Finally, RWD integration combines evidence from commonly used real-world data sources, including patient and device registries, electronic health records, administrative claims databases, and post-market surveillance/follow-up studies, with observational and modelling approaches to complement or substitute clinical trial evidence, particularly when RCTs are difficult or impractical to conduct (15, 25, 27, 30, 35, 36, 38, 40).

**Figure 4:**
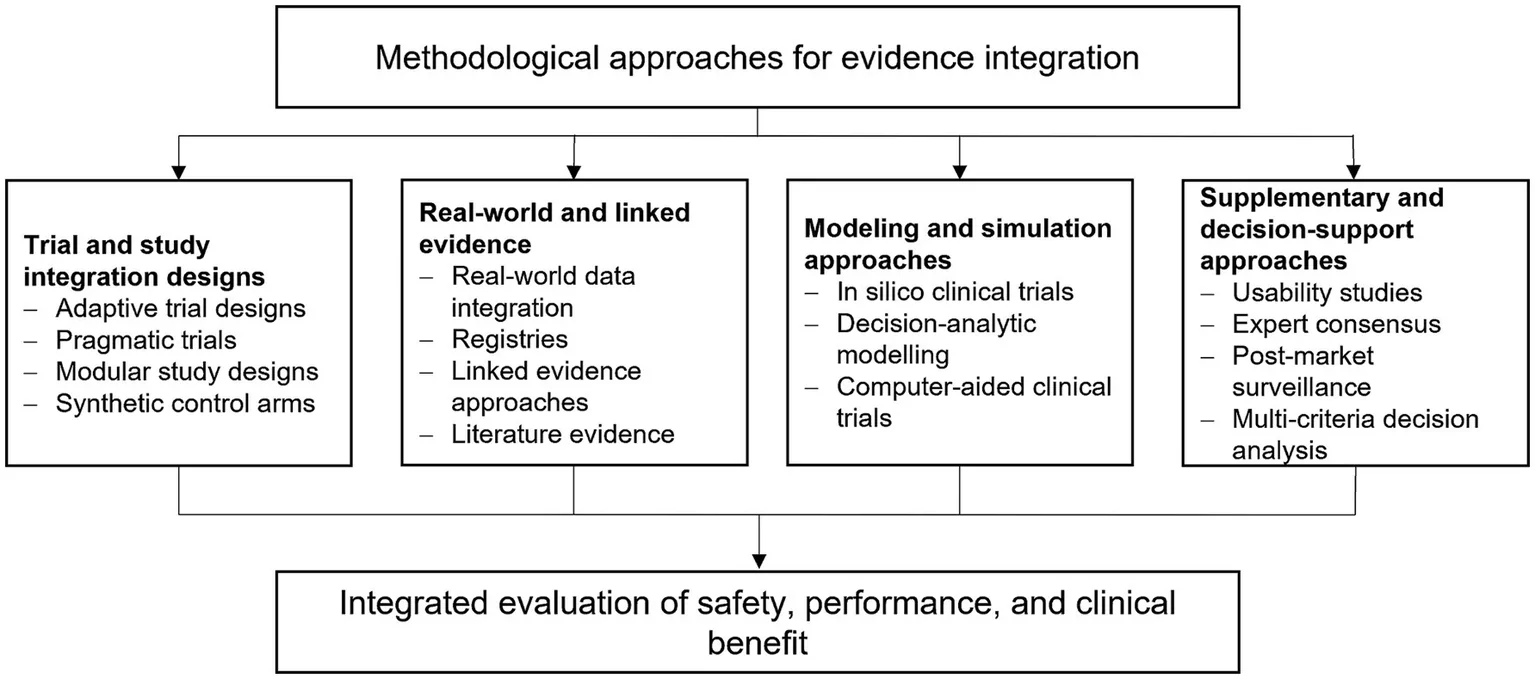
Framework for integrating heterogeneous evidence in high-risk medical devices and *in vitro* diagnostic medical devices.

The identified approaches for integrating evidence were organized into four broad methodological categories: trial and study integration designs, real-world and linked evidence approaches, modelling and simulation approaches, and supplementary and decision-support approaches. Their relationship within integrated evaluation frameworks is illustrated in Figure 4.

#### 3.4.2 Purpose and regulatory application

The integration of different types of evidence serves several important regulatory and practical purposes in the evaluation of high-risk and innovative MDs and IVDs. One of the main purposes is to support a comprehensive benefit–risk assessment. Combining evidence from multiple sources provides a stronger demonstration of safety, performance, and effectiveness while reducing the limitations of relying on a single type of evidence (15, 25, 27–30, 34–37, 39, 41, 42).

Another important purpose is meeting regulatory requirements. Regulatory frameworks such as the EU MDR, EU IVDR, FDA guidance documents, and policies from the Japanese Pharmaceuticals and Medical Devices Agency (PMDA) increasingly encourage or require the use of integrated evidence from both clinical investigations and real-world use, particularly for high-risk and innovative devices (15, 25, 27–30, 34–42).

Integration is also important for addressing device-specific challenges. For devices characterized by rapid innovation, operator dependence, or small patient populations, conventional RCTs may not be feasible. Integrated evidence approaches allow regulatory submissions to better reflect these specific challenges (15, 27, 30, 35, 37, 38, 40–42).

Integration supports conditional approvals and evidence generation throughout the product life cycle. Adaptive frameworks and RWD enable staged approvals and continuous data collection, supporting ongoing reductions in uncertainty and PMS activities (15, 27, 35–38, 42).

#### 3.4.3 Limitations and challenges

Data heterogeneity is a major challenge. Differences in patient populations, study designs, measurement methods, and data quality can make evidence synthesis more difficult and may reduce the comparability and validity of results (15, 27–30, 34–42). The increasing use of real-world data has also highlighted the importance of data quality frameworks and regulatory guidance for assessing the suitability and reliability of these data sources (36).

Uncertainty and bias also remain important concerns. Combining sources with different levels of validity may introduce uncertainty, while managing prior data conflict, confounding, and potential bias is methodologically demanding (15, 27, 29, 30, 34–36, 38–40).

Methodological transparency is essential for regulatory acceptance. Regulatory authorities require clear justification and documentation of integration methods, especially when indirect or surrogate evidence is used (28, 30, 41).

There is also variability in regulatory acceptance across different methodologies. Acceptance of integrated evidence differs between regulators, particularly for newer approaches such as in silico modelling, RWE, and mixed-source meta-analyses. Early communication with regulatory authorities and alignment with evolving standards are therefore important (15, 25, 27, 29, 30, 34–38, 40–42).

Resource and capacity limitations may restrict the wider use of advanced integration methods. Approaches such as MCDA and Bayesian modelling require specialized expertise and infrastructure, which may not be available in all settings (40).

#### 3.4.4 Variability and gaps in the literature

Despite the existence of overarching frameworks, important gaps in methodological guidance remain, particularly for the integration of highly heterogeneous or indirect evidence. This is especially apparent in the evaluation of diagnostic devices and organizational impact, where structured methodological approaches are still not well developed (35, 36, 39, 40, 42).

Regulatory uncertainty also persists, with varying levels of acceptance of integrated evidence types such as RWE, modelling approaches, and mixed-source meta-analyses. Concerns mainly relate to bias, data quality, and methodological transparency (15, 25, 27, 29, 30, 34–38, 40, 42).

Several areas remain insufficiently explored, including the integration of usability evidence, organizational impact data, and complex digital health evidence. These areas are still not fully recognized within formal regulatory frameworks (29, 40, 41).

In addition, challenges related to data standardization and infrastructure are frequently identified as major barriers to effective evidence integration. Fragmented data sources, limited standardization, and insufficient methodological capacity make it difficult to harmonize and implement integration strategies (15, 25, 27–30, 34–40).

## 4 Discussion

The aim of this scoping review was to map and synthesize methodologies for evidence integration and bridging studies relevant to high-risk and innovative MDs and IVDs. Given the increasing complexity of evidence generation under evolving regulatory frameworks, this review sought not only to identify available methodological approaches but also to understand how these methods are being positioned within broader evidence-generation ecosystems. The findings suggest that the field is transitioning from isolated study-based evaluation models toward more integrated, lifecycle-oriented evidence strategies that combine multiple evidence sources across different stages of device development and post-market use.

Evidence generation for MDs and IVDs differs from traditional pharmaceutical development pathways in several important aspects (1, 43). Unlike medicinal products, medical devices often evolve through iterative modifications over their lifecycle, and their performance may be influenced by factors such as operator experience, learning curves, and characteristics of the clinical setting (1, 43, 44). These features can complicate the design and conduct of conventional randomized clinical trials and have prompted discussion on whether evidence frameworks developed for pharmaceuticals can always be directly applied to medical technologies (2, 43, 45).

The findings suggest that evidence integration is increasingly conceptualized as a continuous process rather than a sequence of isolated studies (8, 44). Lifecycle evidence generation frameworks increasingly describe evidence development as an iterative process in which information accumulates from early feasibility investigations through PMS and long-term follow-up (44). Olson proposed integrated evidence generation as a strategic approach in which different evidence sources provide complementary information over time rather than functioning independently (8). The studies identified in this review appear consistent with this perspective and indicate growing recognition that no single study design can adequately address all evidentiary needs associated with high-risk and innovative technologies (2, 43, 45).

The increasing prominence of RWE may reflect broader changes in how evidentiary uncertainty is addressed in high-risk and innovative MDs and IVDs. Rather than serving solely as a source of post-market information, RWE increasingly appears to support evidence integration strategies by complementing limitations of conventional investigations and addressing evidence gaps that may remain after premarket evaluation (11, 17, 35). This shift may be particularly relevant for high-risk devices entering clinical use with limited premarket evidence, where long-term safety and effectiveness often rely on continued post-market assessment (44, 46). Recent EU MDR and EU IVDR frameworks appear consistent with this direction by emphasizing continuous evidence development across the device lifecycle (4–6, 44).

One possible interpretation is that evidence integration is increasingly being used not simply to accumulate more evidence, but to combine complementary forms of information that address different sources of uncertainty. However, increasing reliance on RWE should not be interpreted as a replacement for conventional clinical investigations, as its contribution appears highly dependent on methodological rigor and context (11, 35). Although structured approaches such as coordinated registry networks may strengthen evidence infrastructures (11), unresolved concerns related to data quality, bias, and confounding continue to affect interpretability (35, 36). These findings suggest that methodological consensus on optimal approaches to integrating and interpreting RWE is still developing.

The increasing attention to Bayesian and borrowing approaches may reflect broader efforts to address evidentiary challenges that are difficult to resolve with conventional study designs alone. Rather than functioning solely as statistical techniques, these approaches increasingly appear to represent strategies for incorporating existing knowledge into situations where evidence generation is constrained by limited populations, evolving technologies, or practical barriers to recruitment (15, 16, 26). By allowing the use of prior information from historical studies, external cohorts, or registries, borrowing approaches may help reduce inefficiencies associated with generating entirely new evidence when traditional investigations are difficult to conduct (16, 26). This may be particularly relevant in contexts involving rare diseases or highly specialized populations where generating large randomized datasets remains challenging (15).

Bridging methodologies identified in this review appear to address similar challenges from a different perspective. Rather than combining evidence across multiple sources, bridging approaches seek to extend or transfer existing evidence across populations, settings, or contexts where direct evidence may be unavailable (14, 33). One possible interpretation is that these approaches reflect an increasing recognition that generating complete evidence in every specific context may not always be feasible. Consequently, bridging strategies may provide practical mechanisms to reduce unnecessary duplication of investigations and support more efficient use of available evidence. However, their implementation remains dependent on assumptions about similarity and transferability, which currently lack consistent methodological and regulatory guidance (14, 33).

Taken together, the increasing interest in borrowing and bridging approaches may reflect broader tensions between methodological rigor and practical feasibility. Conventional evidence hierarchies continue to prioritize RCTs, yet such designs may be difficult, expensive, or ethically challenging in certain device contexts (1, 43). Borrowing and bridging methodologies, therefore, appear less as alternatives to rigorous evidence generation and more as attempts to reconcile evidentiary expectations with real-world constraints. However, despite increasing methodological innovation, implementation within routine regulatory practice appears relatively limited, suggesting that methodological development may currently be progressing more rapidly than regulatory operationalization (47, 48).

Recent European regulatory reforms may have influenced not only evidence requirements but also how evidence is generated, integrated, and interpreted for high-risk MDs and IVDs. EU MDR and EU IVDR introduced stronger expectations regarding the quality of clinical evidence and post-market follow-up in response to concerns that some devices entering the market lacked sufficient evidence (3, 5, 6, 45, 46). One possible interpretation is that stricter evidentiary requirements may indirectly encourage greater use of evidence integration approaches, particularly in situations where generating large conventional datasets is difficult. However, stakeholder reports simultaneously describe increasing administrative complexity and uncertainty regarding evidentiary expectations (47, 48). Taken together, these findings suggest that strengthening evidence requirements alone may be insufficient if implementation challenges limit feasibility or create unintended barriers to innovation.

The review also identified distinctive methodological considerations for IVDs that may have implications for evidence integration and bridging strategies. Unlike therapeutic technologies, evidence pathways for diagnostic devices frequently rely on indirect relationships linking analytical performance, diagnostic accuracy, clinical decision-making, and patient outcomes (4, 28, 42). As a result, evidence synthesis in diagnostic contexts may require integrating multiple interconnected components of evidence rather than directly demonstrating clinical benefit alone. This may partly explain why bridging and indirect evidence approaches appear particularly relevant for IVDs.

Emerging digital and data-driven technologies introduce additional complexity and may further challenge traditional evidence paradigms. AI-enabled and software-based devices may continue evolving after implementation, potentially creating tensions between static evidence frameworks and dynamic technologies (10). One possible interpretation is that continuously evolving technologies may require more adaptive evidence models that integrate evidence over time rather than relying on isolated evaluations. Technology-enabled clinical trials and digital infrastructures may represent early attempts to address these challenges, although methodological standards remain under development (49, 50).

The observed heterogeneity across studies may itself represent an important finding. The diversity of evidence sources and study types identified in this review may suggest that evidence integration and bridging methodologies remain conceptually and methodologically fragmented. Rather than reflecting a mature field characterized by standardized approaches, this heterogeneity may indicate an area still undergoing methodological development. The absence of standardized terminology and reporting frameworks further complicates comparison, synthesis, and implementation across studies. The findings also suggest that several methodological areas remain underrepresented in the current literature. In particular, methodological guidance for integrating heterogeneous and indirect evidence, evidence integration for IVDs, digital health technologies, and data standardization remains limited, highlighting important priorities for future methodological development.

Several limitations should be considered when interpreting these findings. Consistent with scoping review methodology, the purpose of this review was to map and characterize available evidence rather than critically appraise methodological quality or formally compare the effectiveness of approaches (12). The intentionally broad inclusion strategy incorporated heterogeneous evidence sources, including grey literature and guidance documents. Although this approach improved comprehensiveness, it also introduced variability in methodological rigor. Also, AI-assisted extraction methods were used to support topic identification across a large and heterogeneous evidence base. Although all outputs underwent human verification and correction against the sources, AI-assisted approaches may introduce risks of omissions or interpretive variability. Iterative prompt development and reviewer oversight were implemented specifically to minimize these risks. An additional limitation is that, although this review defines bridging studies within the broader framework of evidence integration, it did not aim to establish a formal conceptual taxonomy or systematically distinguish bridging from related methodological approaches such as indirect comparisons, dynamic borrowing, extrapolation, or other evidence integration strategies. As a result, some conceptual overlap between these approaches may remain, reflecting the evolving terminology and methodological landscape in the literature.

Overall, the findings suggest that evidence integration and bridging methodologies are becoming increasingly important strategies for addressing evidentiary challenges associated with high-risk and innovative MDs and IVDs. Rather than replacing conventional investigations, these approaches increasingly serve as mechanisms to combine, extend, and contextualize evidence from heterogeneous sources. However, despite substantial methodological innovation, standardization and regulatory implementation remain limited.

## 5 Conclusion

This scoping review identified a growing range of methodologies for comparing and integrating evidence to evaluate high-risk and innovative medical devices and *in vitro* diagnostic medical devices. Approaches such as Bayesian methods, adaptive trial designs, real-world data integration, linked evidence, and bridging studies are increasingly used to address limitations of conventional clinical trials. Despite growing acceptance of these approaches, important challenges remain, including heterogeneity of evidence, methodological complexity, limited standardization, and variability in regulatory acceptance. Evidence gaps were particularly evident for in vitro diagnostic medical devices, digital technologies, and indirect evidence integration. Further methodological harmonization, transparent reporting, and clearer guidance are needed to support the reliable and consistent integration of evidence across the medical device life cycle.

## Data Availability

The original contributions presented in the study are included in the article/Supplementary material, further inquiries can be directed to the corresponding author.
