# Supplementary material for: Evidence integration and bridging methodologies for high-risk and innovative medical devices and in vitro diagnostic medical devices: a scoping review
Source: Front Med (Lausanne). 2026 Aug 28;13:1917208. doi: 10.3389/fmed.2026.1917208 (PMC13561784; doi:10.3389/fmed.2026.1917208)
Supplement: Supplementary file 2 [file Supplementary_file_2.docx]

**Evidence Integration and Bridging Methodologies for High-Risk and Innovative Medical Devices and In Vitro Diagnostic Medical Devices: A Scoping Review**

**Prompts used in pilot testing**

I. Methods for comparing evidence

*“Please review the following scientific article and provide a concise narrative summary of what the article explicitly states about methods for comparing clinical and/or performance evidence in the context of meeting regulatory requirements for high-risk or innovative medical devices (MDs) and in vitro diagnostic devices (IVDs). Include only information that directly describes such comparative methods as discussed in the article. For example, such methods could be meta-analyses, indirect comparisons, network meta-analyses, bridging studies, or comparative effectiveness research. Focus solely on how these methods are described, applied, or recommended for assessing safety, performance, or benefit-risk profiles in support of regulatory submissions. Do not include any information that is not explicitly related to methods for comparing evidence. The summary must be based only on what is clearly stated in the article, without interpretation or expansion. Write clearly, concisely, and with emphasis on methodological aspects relevant for regulatory decision-making. Write in a connected narrative format, not as bullet points.”*

II. Integration of different types of evidence

*“Review the following scientific article and summarise what it explicitly states about the integration of different types of clinical and performance evidence to support regulatory requirements for high-risk or innovative medical devices (MDs) and in vitro diagnostic devices (IVDs). Focus on how evidence from diverse sources (e.g., clinical trials, real-world data, preclinical studies, literature, expert opinion, or modelling, etc.) is combined or synthesised. Describe any frameworks, methodologies, or best practices used or recommended for integrating this evidence, especially in support of benefit-risk assessment, conformity assessment, or regulatory submissions. Mention any discussion on how integration contributes to a more robust or comprehensive evaluation, and note challenges such as data heterogeneity, uncertainty, or regulatory acceptance. The summary must be based only on what is clearly stated in the article, without interpretation or expansion. Write clearly, concisely, and with emphasis on methodological aspects relevant for regulatory decision-making. Write in a connected narrative format, not as bullet points.”*

**Mapping and synthesis prompts**

I. Methods for comparing evidence

*“You are given a collection of excerpted texts from multiple scientific studies, each labelled by Study ID (such as "Study ID #4"). All excerpts relate specifically to methods for comparing clinical and/or performance evidence in the context of fulfilling regulatory requirements for high-risk or innovative medical devices (MDs) and in vitro diagnostic devices (IVDs).*

*Your task is to synthesise this material into a comprehensive, structured, and analytical report addressing the following:*

*Overview of comparison methods: Identify and describe the various methods for comparing evidence that are discussed across the studies. Clarify how these methods are characterised and in what contexts they are used or recommended. Methods may include, but are not limited to, meta-analyses, indirect comparisons, network analyses, bridging studies, or structured comparison frameworks.*

*Purpose and application in regulatory context: Explain the intended use of these methods in generating regulatory evidence. Describe how they are applied to assess safety, performance, effectiveness, equivalence, or benefit-risk profiles, and how they support regulatory submissions or conformity assessment.*

*Strengths, limitations, and challenges: Summarise any advantages, methodological limitations, or challenges reported in relation to the comparability of evidence across different study types, populations, or device categories.*

*Regulatory considerations: Present any explicit discussion of how these comparative methods are viewed by regulators, including any references to MDR or IVDR, and relevant regulatory expectations, flexibilities, or constraints.*

*Variability and gaps across the literature: Identify any inconsistencies, variations in depth or clarity, conflicting perspectives, or underexplored areas across the included texts related to comparative methodologies.*

✅ *Write in a clear academic narrative, using appropriate headings and subheadings.*

🚫 *Do not summarise each study separately. Focus on synthesising content thematically.*

🚫 *Do not include any information that is not explicitly present in the source texts.”*

II. Integration of different types of evidence

*“You are given a collection of excerpted texts from multiple scientific studies, each labelled by Study ID (such as "Study ID #4"). All excerpts relate specifically to the integration of different types of clinical and/or performance evidence in the context of fulfilling regulatory requirements for high-risk or innovative medical devices (MDs) and in vitro diagnostic devices (IVDs).*

*Your task is to synthesise this material into a comprehensive, structured, and analytical report addressing the following:*

*Overview of integration approaches: Identify and describe the various methods or strategies for integrating different types of evidence discussed across the studies. Explain how integration is conceptualised or operationalised, and what types of evidence are typically combined (such as clinical trial data, real-world evidence, registries, literature, expert input, or modelling).*

*Purpose and application in regulatory context: Describe the rationale for integrating multiple types of evidence and how this contributes to regulatory evidence generation. Explain how integration supports the demonstration of safety, performance, effectiveness, or benefit-risk profiles in regulatory submissions.*

*Strengths, limitations, and challenges: Summarise any advantages, methodological complexities, or challenges associated with integrating diverse evidence types, such as issues of heterogeneity, data compatibility, uncertainty, or methodological transparency.*

*Regulatory considerations: Present any explicit discussion of how integrated evidence is treated within regulatory frameworks, including references to MDR or IVDR, and any regulator expectations, flexibilities, or concerns.*

*Variability and gaps across the literature: Identify any inconsistencies, variation in terminology or implementation, conflicting perspectives, or underexplored areas related to evidence integration in the included texts.*

✅ *Write in a clear academic narrative, using appropriate headings and subheadings.*

🚫 *Do not summarise each study separately. Focus on synthesising content thematically.*

🚫 *Do not include any information that is not explicitly present in the source texts.”*
